# Association Between Childhood Maltreatment and Symptoms of Obsessive-Compulsive Disorder: A Meta-Analysis

**DOI:** 10.3389/fpsyt.2020.612586

**Published:** 2021-01-20

**Authors:** Wenwen Ou, Zhijun Li, Qi Zheng, Wentao Chen, Jin Liu, Bangshan Liu, Yan Zhang

**Affiliations:** ^1^Department of Psychiatry, The Second Xiangya Hospital, Central South University, Changsha, China; ^2^Hunan Key Laboratory of Psychiatry and Mental Health, China National Clinical Research Center on Mental Disorders (Xiangya), China National Technology Institute on Mental Disorders, Hunan Technology Institute of Psychiatry, Mental Health Institute of Central South University, Changsha, China; ^3^Department of Psychiatry, Xianyue Psychiatric Hospital, Xiamen, China

**Keywords:** OCD, childhood maltreatment, meta-analysis, association, clinical symptomatology

## Abstract

**Background:** Previous studies have indicated that childhood maltreatment (CM) may potentially influence the clinical symptomatology of obsessive-compulsive disorder (OCD). Here, we aimed to quantify the relationship between CM and obsessive-compulsive symptoms (OCS) and depressive symptoms in OCD through a meta-analysis.

**Method:** We searched PubMed, Embase, Cochrane Library, and PsycARTICLES databases for articles reporting the association between CM and OCD on April 15, 2020. Random-effect models were used to quantify the relationship between CM and the severity of OCS and depressive symptoms in OCD.

**Results:** Ten records with 1,611 OCD patients were included in the meta-analysis. The results revealed that CM is positively correlated with the severity of OCS [*r* = 0.10, 95%Confidence Interval (CI): 0.01–0.19, *P* = 0.04] as well as depressive symptoms in OCD (*r* = 0.15, 95%CI: 0.07–0.24, *P* = 0.0002). For the subtypes of CM, childhood emotional abuse (CEA) and childhood sexual abuse (CSA) was related with the severity of OCS (*r* = 0.11, 95%CI: 0.03–0.19, *P* = 0.009) and obsession (*r* = 0.13, 95%CI: 0.03–0.23, *P* = 0.01), respectively.

**Conclusion:** Our meta-analysis indicates that OCD patients who suffered more CM may exhibit more severe OCS and depressive symptoms.

## Introduction

Obsessive-compulsive disorder (OCD) is an impairing, chronic mental disorder characterized by obsessions or compulsions. Obsessions often refer to recurrent, intrusive, and contradictory thoughts or impulsive intentions. Compulsions mostly consist of repetitive, ritual, or pathological behaviors, thereby reducing anxiety and depression caused by the obsessions. OCD exerts significant social and occupational impairment to the sufferers (1, 2). Moreover, about 55% of OCD patients have psychiatric comorbidities (3, 4). According to the World Health Organization (WHO) (5), OCD ranks among the top 10 disabling diseases. In China, the lifetime and 12-months prevalence of OCD in China are as high as 2.4 and 1.6%, respectively (6), resulting in a significant burden to the Chinese population.

Childhood maltreatment (CM) refers to the abuse and neglect suffered by individuals younger than 18 years. There are five types of CM: childhood physical abuse (CPA), childhood emotional abuse (CEA), childhood sexual abuse (CSA), childhood physical neglect (CPN), and childhood emotional neglect (CEN) (7). It is proposed that maltreatment in childhood may be associated with an increased risk of developing psychiatric disorders (such as OCD) in later life (8, 9). Besides, considerable studies have reported that OCD patients report significantly more CM when compared with matched healthy controls (HCs) (10–15). Notably, there are several studies based on population or clinical sample claiming that CM is associated with the severity of obsessions or compulsions in OCD (15–17).

As is well-known, studies of comorbidity in OCD have reported that OCD sufferers are often accompanied by a high level of depressive symptoms (2–4, 18). A clinical study that enrolled 160 patients diagnosed with OCD found a higher depressive level in the childhood trauma (CT)-exposed group than non-CT exposed group (19). Moreover, empirical studies have pointed out the unique relationship between the CM and the severity of depressive symptoms in OCD (19, 20).

Despite the above intriguing findings, there are also inconsistent results. For instance, a clinical study investigating the association between CM and obsessive-compulsive symptoms (OCS) severity has revealed a non-significant effect of CM on OCS (21). Subsequently, another cross-sectional study based on Netherlands Obsessive Compulsive Disorder Association (NOCDA) was in agreement with the above conclusion (22). Meanwhile, the results of studies in 67 patients with OCD showed no significant difference in the severity of depressive symptoms between two groups: patients who have experienced ACE and those who do not (23).

Since the specific relationship between CM and symptoms of OCD is poorly understood, we performed the meta-analysis to quantify the magnitude and significance of correlations between CM and OCS severity in patients with OCD and quantitatively summarize the association of CM and the severity of depressive symptoms in OCD patients.

## Methods

### Search Strategy and Selection Criteria

We searched PubMed, Embase, Cochrane Library, and PsycARTICLES databases for the articles exploring the association of CM with the severity of OCS and depressive symptoms in OCD. The references of relevant studies were subject to hand searching. The search was conducted on April 15, 2020 by the following search terms: “child^*^ abuse,” “child^*^ neglect,” “child^*^ maltreatment,” “child^*^ adversity,” “child^*^ trauma,” “sexual abuse,” “physical abuse,” “emotional abuse,” “physical neglect,” “emotional neglect,” “early experience,” “early interpersonal trauma,” “early abuse,” “early maltreatment,” and “early neglect” for CM, combined with “Obsessive-compulsive disorder,” “Obsessive-compulsive disorder,” “Obsessive-compulsive neurosis,” and “OCD” for OCD. This study was prospectively registered at https://www.crd.york.ac.uk/prospero~(CRD42020179565).

We identified articles satisfying the following criteria: (1) studies quantitatively assessed CM history, OCS severity, as well as the severity of depressive symptoms in OCD. CM should be defined as the exposure to CPA, CEA, CSA, CPN, and CEN before 18 years old; (2) studies quantitatively assessed the relationship between CM and OCS or depressive symptoms, either by correlation analysis or by *t*-test of the difference between those with CM and those without CM; (3) studies should be published in English. Studies were excluded if they were: (1) qualitative studies, such as case reports and reviews; (2) studies with no available data for data synthesis.

### Data Extraction

Information was extracted by two independent reviewers (ZL and QZ) and imported into an excel worksheet (Excel for MacOS, 2016). Inconsistencies were settled by consensus meetings. The following information was obtained from eligible studies: (1) sample characteristics: age, sample size, diagnostic criteria; (2) study characteristics: study design, CM measurement, and CM types, measurement of OCS or depressive symptoms in OCD; (3) primary outcome: the correlation coefficient between CM and OCS and depressive symptoms in OCD patients, or the standardized mean difference in OCS or depressive symptoms between those with CM and those without CM. Besides, authors were contacted if any important information is missing or incomplete.

### Quality Assessment

The quality of case-control studies was examined by the Newcastle Ottawa Scale (NOS), which was recommended by the Cochrane Collaboration (24). Studies coring ≥7 were considered high-quality studies, while studies coring <7 were considered low-quality studies (25). The quality of cross-sectional studies was assessed by an 11-item checklist, which was approved by the Agency for Healthcare Research and Quality (AHRQ) (26). Studies scoring 0–3, 4–7, and 8–11 were interpreted as low, moderate, and high quality, respectively (27).

### Data Synthesis and Analysis

Extracted data were uniformly converted to Pearson correlation coefficients (r_*p*_) for data synthesis. In articles where Spearman correlation coefficients (r_*s*_) were reported, the r_*s*_*s* was converted to r_*p*_*s* using the formula r_*p*_ = 2sin(r_*s*_$\frac{\pi }{6}$) (28, 29). Similarly, in articles where continuous data [mean or standard deviations (SDs)] was reported, the means and SDs were transformed in r_*p*_s using the following methods. Firstly, the standardized mean difference (SMD) was calculated by the mean difference in OCS between the maltreated and non-maltreated OCD groups divided by the pooled SD. Then, the SMDs were transformed to r_*p*_s according to the formula *r* = $\frac{SMD}{\sqrt{SMD^{2}+A}}$ (A refers to values related to sample size) provided by Cooper and Hedges (30).

The analytical work was conducted by Review Manager (version 5.3 for MacOS) and Excel 2016. Firstly, all of the r_*p*_s were converted to Fisher's Z for normalization. Then, the summary effect sizes and confidence intervals were calculated using the value of Fisher's Z and its standard error (SE). Finally, we converted the above values back to r_*p*_ for interpretation. The transformation formula between r_*p*_ and Fisher's Z was presented as follows: (1) Fisher's Z = 0.5 × $\ln\frac{1+r}{1-r}$; (2) Vz = $\frac{1}{n-3}$ (the variance of Z); (3) SE = $\sqrt{\;Vz}$; (4) summary r$=\frac{e^{2z}-1}{e^{2z}+1}\;$(z refers to summary Fisher's Z) (30). According to Cohen's guidelines (31), a r_p_ 0.1–0.3, 0.3–0.5, and ≥0.5 suggests a small, medium, and large correlation coefficient, respectively.

Considering the substantial variation in the study design of included studies, random-effect models were selected for data synthesis. Heterogeneity across the studies was evaluated by the chi-square and I-square statistics. *P* < 0.1 in the chi-square statistic indicates significant heterogeneity across the studies (32). The I^2^ statistics reflect the percentage of total variation across studies due to heterogeneity rather than sampling error, with the values of 25, 50, and 75% indicating low, moderate, and high heterogeneity (33). Subgroup analyses were performed to identify the potential factors, such as sample size and assessment tools for CM, which may influence the association between the CM and the clinical symptoms of OCD. Similarly, sensitivity analyses were conducted to identify the relative effects of individual studies on the pooled effect size by sequentially removing one study and reanalyzing the remaining datasets. Finally, funnel plots were adopted to assess publication bias. Significance was set as a two-tailed *P* < 0.05 for all of the analyses.

## Results

### Literature Search and Screening

**Figure 1:**
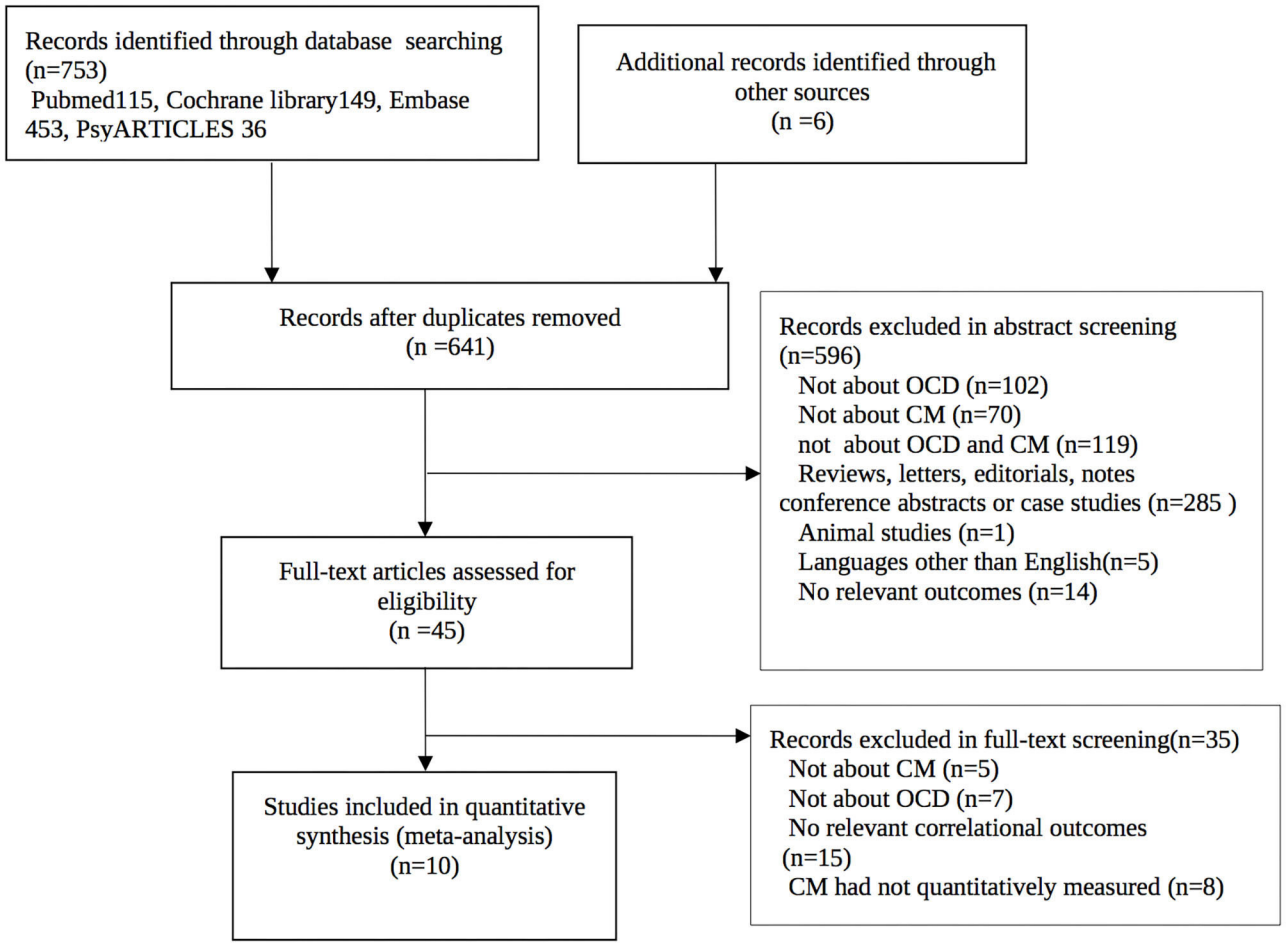
Study selection procedure.

The initial search retrieved 759 records with 118 duplicates. Five hundred and ninety-six records were excluded in the title and abstract screening step. Thirty-five records were further excluded in the full-text screening step. Finally, ten records with 1,611 OCD patients were included in the meta-analyses. The process of the literature search and screening is presented in Figure 1.

### Characteristics of the Included Studies

All of the ten included studies (19–21, 23, 34–39) employed a cross-sectional design except for Wang et al. (39) and Bey et al. (20), which employed a case-control design. All studies used the Yale Brown Obsessive Compulsive Scale (YBOCS) to evaluate the severity of the OCS in OCD patients. Seven studies used the Childhood Trauma Questionnaire (CTQ) to assess the severity of CM. The other three studies [Benedetti et al. (37), Semiz et al. (34), and Wang et al. (39)] used the Risky Families Questionnaire (RFQ), Traumatic Experiences Checklist (TEC), Early Trauma Inventory Self Report-Short Form (ETISR-SF), respectively for the assessment of CM. The quality of the included studies is low to moderate, ranging from 2 to 7 in AHRQ or NOS. The main characteristics and quality assessment of the included studies are described in Table 1.

**Table 1:** Characteristics of included studies.

**Study ID**	**Region**	**Sample size (*N*)**	**Design**	**Diagnostic criteria**	**OCD measure**	**CTQ measure**	**Depressive symptoms measure**	**NOS or AHRQ**
Ay and Erbay (23)	Turkey	67	Cross-sectional	DSM-5	YBOCS	CTQ-28	BDS	5
Kart and Türkçapar (19)	Turkey	160	Cross-sectional	DSM-IV	YBOCS	CTQ-28	BDI	6
Benedetti et al. (37)	Italy	40	Cross-sectional	DSM-IV	YBOCS	RFQ	N/A	4
Semiz et al. (34)	Turkey	120	Cross-sectional	DSM-IV	YBOCS	TEC	BDI	7
Selvi et al. (21)	Turkey	95	Cross-sectional	DSM-IV	YBOCS	CTQ-28	BDI	3
Bey et al. (20)	Germany	169	Case-control	DSM-IV	YBOCS	CTQ	BDI-II	7
Krah and Koopmans (35)	Netherlands	281	Cross-sectional	DSM-IV-TR	YBOCS	CTQ	BDI-II	5
Carpenter and Chung (38)	United Arab Emirates	89	Cross-sectional	N/A	YBOCS	CTQ-R	N/A	2
Coban and Tan (36)	Turkey	106	Cross-sectional	DSM-5	YBOCS	CTQ	HAMD	5
Wang et al. (39)	China	484	Case-control	DSM-IV	YBOCS	ETISR-SF	BDI	6

*YBOCS, Yale Brawn Obsessive-Compulsive Scale; CTQ, Child Trauma Questionnaire; CTQ-R, Child Trauma Questionnaire-Revised; RFQ, Risk Families Questionnaire; TEC, Traumatic Experience Checklist; ETISR-SF, Early Trauma Inventory Self- Report-short Form; BDS, Beck Depression Scale; BDI, Beck Depression Inventory; BDI-II, Beck Depression Inventory-II; HAMD, Hamilton Rating Scale for Depression; MDD, major depressive disorder; ADHD, attention deficit and hyperactivity disorder; N/A, not available*.

### Relationship Between CM and Severity of OCS and Depressive Symptoms

**Figure 2:**
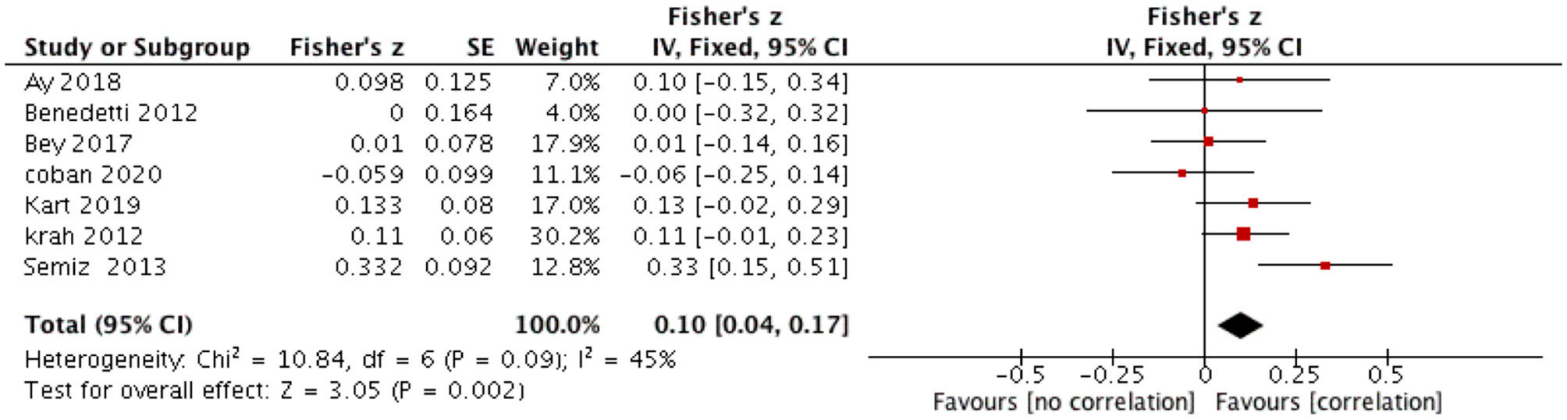
Correlation between CM and total severity of OCS.

The relationship between CM and severity of OCS in OCD was reported in seven records with 943 participants. Random-effect models showed that CM has a weak but significant correlation with the severity of OCS (summary Fisher's Z = 0.10, 95%CI: 0.01–0.19, r_*p*_ = 0.10, *P* = 0.04) (Figure 2). The correlation was weak. There was moderate heterogeneity across the included studies (*x*^2^ = 10.84, I^2^ = 45%, *P* = 0.09). The associated Funnel Plot was approximately symmetrical, suggesting that the possibility of publication bias is low (Supplementary Material).

**Figure 3:**
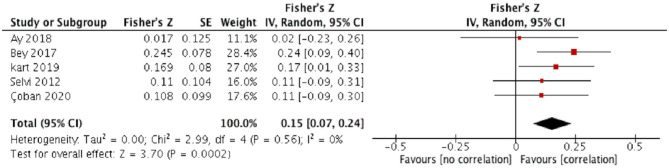
Correlation between CM and severity of depressive symptoms in OCD.

The relationship between CM and severity of depressive symptoms was tested using five records, with 597 participants. Random-effect models showed that CM positively correlates with the severity of depressive symptoms (summary Fisher's Z: 0.15, 95%CI: 0.07–0.24, r_*p*_ = 0.15, *P* = 0.0002) (Figure 3). Heterogeneity across studies was low (*x*^2^ = 2.99, I^2^ = 0%, *P* = 0.56), indicating that the result was relatively stable. The associated Funnel Plot was approximately symmetrical (Supplementary Material).

### Relationship Between CM Subtypes and Severity of OCS

The results of the relationship between CM subtypes and OCS severity were summarized in Table 2. For the severity of OCS, random-effect models revealed a positive relationship between CEA and the total OCS severity (summary Fisher's Z: 0.11, 95%CI: 0.03–0.19, r_*p*_ = 0.11, *P* = 0.009), with moderate heterogeneity across the included studies. No significant correlation was found between CPA, CSA, CPN, and OCS severity. For OCS dimensions (including the obsession and compulsion), random-effect models showed that SA correlates with obsession (summary Fisher's Z: 0.13, 95%CI: 0.03–0.23, *P* = 0.01), while CPA, CEA, and CEN did not correlate to obsession and compulsion. The forest plots of the above meta-analyses were presented in Supplementary Material.

**Table 2:** The association between the subtype of CM and OCS severity.

**Subtype**	**Studies (*n*)**	**Sample size (*n*)**	***X*^**2**^**	**Heterogeneity I^**2**^**	***p***	**Effect size**	**Summary fisher's Z 95%CI**	***p***	**Rp**
**Obsession**									
CEA	5	994	10.85	63%	0.03	0.13	0.00–0.25	0.05	0.13
CPA	4	510	15.88	81%	0.001	0.06	−0.10–0.23	0.46	0.06
CSA	4	510	6.19	52%	0.10	0.13	0.03-0.23	0.01*	0.13
CEN	4	510	29.25	90%	<0.000001	0.13	−0.09–0.25	0.25	0.13
**Compulsion**									
CEA	4	510	9.82	69%	0.22	0.11	−0.02–0.23	0.11	0.11
CPA	4	510	12.33	76%	0.006	0.03	−0.11–0.18	0.64	0.03
CSA	4	510	13.76	78%	0.003	0.07	−0.08–0.22	0.37	0.07
CEN	4	510	25.52	88%	<0.0001	0.13	−0.08–0.33	0.23	0.13
**Total**									
CEA	6	1246	8.84	43%	0.12	0.11	0.03–0.19	0.008*	0.11
CPA	5	762	16.88	76%	0.002	0.01	−0.15–0.17	0.92	0.01
CSA	5	762	14.52	72%	0.006	0.09	−0.05–0.23	0.21	0.09
CPN	5	624	0.71	0%	0.87	−0.03	−0.11–0.05	0.45	−0.03
CEN	5	762	23.07	83%	0.0001	0.12	−0.06–0.29	0.18	0.12

CEA, emotional abuse; CPA, physical abuse; CSA, sexual abuse; CPN, physical neglect; CEN, emotional neglect; Total, Total severity of OCS; X^2^, chi-square statistics; I^2^, I-square statistics; R_p_, Pearson correlation coefficients; *P < 0.05. Bold values indicates statistical significance.

### Subgroup and Sensitivity Analyses

Subgroup analyses showed that the variation in CM measurement did not associate with a change in effect size across the meta-analysis. However, a strong association was observed in a relatively larger sample size group than the smaller sample size group. The results are shown in Appendix in Supplementary Material.

Sensitivity analyses revealed that the total heterogeneity of the meta-analysis was reduced when removing the study of Semiz et al. (34) or Coban et al. (36), with the I^2^ reduced to 0 and 37%, respectively.

## Discussion

To the best of our knowledge, this is the first meta-analysis investigating the association between CM and the clinical symptomatology of OCD. Our results revealed that CM positively correlates with the severity of OCS as well as depressive symptoms. Specifically, CEA is correlated with the severity of OCS, and CSA is correlated with obsession. Our findings highlight the significance of CM's role in the symptomatology in OCD.

In line with a growing body of studies, our findings showed that CM was closely related to OCS severity in OCD patients (17, 28, 40). As we well-known, early childhood experience has a profound effect on suffers that results in psychosocial, emotional, and cognitive dysfunction, and the latter correlates with the development of psychiatric disorders or aggravates its underlying vulnerabilities (41). Specifically, current cognitive models for OCD proposed that maladaptive beliefs initially formed as adaptive coping methods with the early childhood experience may later gain obsessive characteristics and finally turn into psychopathology (42). Namely, early childhood experience could induce the emergence of intrusive and unwanted thoughts, which eventually developed into clinical obsessions and compulsions. Moreover, it is well-established that early traumatic events could also increase the frequency and impact content of intrusive thoughts (43). Additionally, two studies conducted by Briggs et al. (16) and Kroska et al. (44) have described that individuals who have experience of CM appear to adopt negative coping styles, which had been proved to function as a mediator in the association between CM and OCS severity in OCD patients. A maladaptive coping strategy, typically defined as an attempt to withdraw when facing the stressor or a belief of inability to deal with the situation, was proved to bring about more severe distress and intensify the severity of OCS (45).

Importantly, our results show that CEA and CSA are positively related to OCS compared to the other subtypes of CM, which also stand in line with the previous epidemiological (46–48) and clinical studies (14, 39, 49). On the one hand, it seems that CSA may have the most damaging psychological impact on a significant proportion of victims after experiencing early traumatic events (50). The CSA victims may experience sustainable disgust beyond the peritraumatic period, so the victims may be mentally disturbed by the sustainable reminder of the abused experience, which was significantly related to OCS (51, 52). The notion was confirmed by two population-based studies, which revealed that CSA correlates with a wide range of psychiatric disorders (such as OCD) in adulthood (47, 53). On the other hand, it is hypothesized that comparing with the other types of CM, CEA may modulate the cognitive style deleteriously. In other words, individuals who have been subject to CEA may tend to develop a negative cognitive style (54), which may link to the later development of OCD. Finally, studies found that the individuals who have the history of early traumatic experience (particularly CEA and CSA) appear to display maladaptive coping strategies that have reported to act as a mediator in the relationship between CM and OCS (44). An emerging study exploring the effects of CM and coping styles on OCS in patients with psychotic disorders has revealed that patients with OCS report more common CEA and CSA than those without OCS. The study further found that patients who have experienced CEA and CSA show a higher preference to adopt negative and avoidant coping styles (55).

In our study, we demonstrated that CM is related to the severity of depressive symptoms in OCD patients. The finding is also consistent with previous studies. Recently, childhood may be described as a critical period for emotional development, since self-emotional regulation develops rapidly in this period (56). Hence, emotion regulation is more likely to subject to several environmental factors (57). Early traumatic experience, one of the acquired environmental factors, has been reported to be associated with emotional dysregulation, which might precipitate the occurrence of affective symptoms (56). For instance, meta-analytic findings found that individuals exposed to CM exhibit more severe depressive symptoms than non-maltreatment controls (58, 59). Other than the environmental factors mentioned, gene-environment interaction also plays a critical role. Studies have implied that the progranulin (PGRN), an element expressed in microglia and neurons that regulates inflammation, is associated with mood regulation in OCD patients (60). Furthermore, updated evidence comes from a study on the Chinese OCD cohort that has proved that the interaction between early traumatic experience and the PGRN gene in the hypothalamus might play an essential role in promoting depressive symptoms in OCD patients (39).

Finally, we did the sensitivity analysis of the association between CM and OCS severity in OCD. The total heterogeneity has reduced significantly by removing the Semiz et al. study and the Coban et al. study (36) in turn. Two reasons may be responsible for these findings: firstly, the OCD sample enrolled in Semiz et al. (34) includes a part of treatment-resistant patients, so the relationship between CM and OCS severity may be influenced by treatment outcomes of the OCD patients. Secondly, the impact of CM on OCS severity was indirect in the Coban et al. study (36), which was found to be influenced by confounding factors, such as comorbidity.

## Limitations

Some limitations should be considered when interpreting our findings. Firstly, since CM was retrospectively assessed by self-report questionnaires in most of the included studies, it is possible that the results may be subject to recall bias, leading to an overestimation or underestimation of the relationship between CM and OCS and depression severity. Secondly, there was substantial heterogeneity in the meta-analysis for the association between the subtypes of CM and OCS severity; however, the source of heterogeneity across the studies cannot be further explored since the number of included studies is relatively low. Thus, the results should be interpreted with caution. Thirdly, we merely included English papers, it is possible that the exclusion of Non-English papers may lead to incomplete inclusion of literature, and the results may be subject to selection bias. Fourthly, the association between CM and OCD severity may be susceptible to many confounders, such as the genetic variation and gene-environment interaction. We are unable to assess the effect of these confounders on the results in our study. Finally, as our meta-analysis is mostly based on cross-sectional data, we are unable to make a causal reference about the relationship between CM and OCD symptomatology, which should be settled by future longitudinal cohort studies.

## Conclusions

This study quantitatively summarized the current evidence about the relationship between CM and clinical symptomatology in OCD. Our findings revealed a close relationship between CM (especially CEA and CSA) and the clinical symptomatology (OCS and depressive symptoms) of OCD. The influence of CM on the clinical symptoms of OCD is small but significant, indicating that we need calls more attention to CM in the assessment and management of OCD. Specifically, the assessment of CM may help predict the outcome of OCD and psychotherapies involving CM intervention may help alleviate OCD symptoms. Nevertheless, we cannot draw a direct causal relationship, given that the most included studies analyzed in our studies are cross-sectional. Hence, future studies are necessary to incorporate prospective or cohort studies to assess the possible causality and temporal relationship between CM and its subtypes and the unfavorable outcomes of OCD. Moreover, the mechanisms mediating the effect of CM and OCD development and symptomatology remain unclear, requiring further investigation.

## Data Availability Statement

The original contributions presented in the study are included in the article/Supplementary Material, further inquiries can be directed to the corresponding author/s.

## Author Contributions

WO conducted statistical analysis, drafted the manuscript edited and submitted the manuscript. ZL and QZ participated in the literature search, study selection and data extraction. WC participated in the literature search and study selection. BL and JL conceptualized and designed the study, critically reviewed and revised the manuscript. YZ conceptualized and designed the study. All authors have approved the final version of this manuscript.

## Conflict of Interest

The authors declare that the research was conducted in the absence of any commercial or financial relationships that could be construed as a potential conflict of interest.
