# Supplementary material for: Association Between Childhood Maltreatment and Symptoms of Obsessive-Compulsive Disorder: A Meta-Analysis
Source: Front Psychiatry. 2021 Jan 20;11:612586. doi: 10.3389/fpsyt.2020.612586 (PMC7854900; doi:10.3389/fpsyt.2020.612586)
Supplement: Supplementary file 1 [file Table_1.DOCX]

**Supplementary Material**

**1. Literature search and screening:**

- 1. **Electronic databases searching:**

**(1) Pubmed:**

#1 (child* abuse[Text Word] OR child* neglect[Text Word] OR child* maltreatment[Text Word] OR child* adversity[Text Word] OR child* trauma[Text Word] OR sexual abuse[Text Word] OR physical abuse[Text Word] OR emotional abuse[Text Word] OR physical neglect [Text Word] OR emotional neglect[Text Word] early experience[Text Word] OR early interpersonal trauma[Text Word] OR early abuse[Text Word] OR early maltreatment[Text Word] OR early neglect[Text Word] **47885 items**

#2 (Obsessive compulsive disorder [Text Word] OR Obsessive-compulsive neurosis [Text Word] OR obsessive-compulsive disorder OR OCD [Text Word] OR Anankastic Personality [Text Word]) **19963 items**

#1 AND #2  **115 items**

**(2) Cochrane library：**

#1 MeSH Child* Abuse OR child* neglect OR child* adversity OR child* trauma OR child* maltreatment OR sexual abuse OR physical abuse OR emotional abuse OR physical neglect OR emotional neglect OR early experience OR early interpersonal trauma OR early abuse OR early neglect OR early maltreatment **40816 items**

#2 MeSH descriptor: Obsessive-Compulsive Disorder OR obsessive-compulsive neurosis OR obsessive compulsive disorder OR OCD OR anankastic personality **2986 items**

#1 AND # 2 **149 items**

**(3) Embase:**

#1 child* abuse/exp OR child* neglect OR child* adversity OR child* trauma OR sexual abuse OR physical abuse OR emotional abuse OR physical neglect OR emotional neglect OR early experience OR early interpersonal trauma OR early abuse OR early neglect OR early maltreatment **58556 items**

#2 obsessive compulsive disorder/exp OR obsessive-compulsive neurosis OR obsessive-compulsive disorder OR OCD OR anankastic personality **44476 items**

#1 AND #2 **453 items**

**(4) PsycARTICLES：**
S1 child* abuse OR child* neglect OR child* maltreatment OR child* adversity OR child* trauma OR sexual abuse OR physical abuse OR emotional abuse OR physical neglect OR emotional neglect OR early experience OR early interpersonal trauma OR early abuse OR early maltreatment OR early neglect  **17777items**

S2 Obsessive-compulsive disorder OR Obsessive-compulsive neurosis OR Obsessive compulsive disorder OR OCD OR Anankastic Personality **3879 items**

S1 AND S2 **36 items**

**1.2 Hand searching: 6 papers were attached from hand searching from references of relevant studies**

**Total items searched from databases: 115+149+453+36+6=759**

The study selection procedure was shown in Fig 1 in the main text.

**2. Quality assessment**

2.1 Quality ratings for the 2 case control studies included on the basis of Newcastle-Ottawa quality assessment scale

|  | **Selection**  (score) |  |  |  | **Comparability**  (score) | **Exposure**  (score) |  |  | **Total Score** |
| --- | --- | --- | --- | --- | --- | --- | --- | --- | --- |
|  | Case definition | Representative of cases | Selections of controls | Definition of controls | Control for Age or Obesity or Smoking or Exercise | Ascertainment of exposure | Same method of ascertainment for participants | Nonresponse rate |  |
| Bey et al. 2017 | 1 | 0 | 1 | 1 | 1 | 1 | 1 | 1 | 7(high) |
| Wang et al. 2020 | 1 | 0 | 1 | 1 | 2 | 0 | 1 | 0 | 7(high) |

2.2 Quality ratings for the 8 cross-sectional studies included on the basis of 11-item checklist of Agency for [Healthcare Research](http://libdb.csu.edu.cn/topics/medicine-and-dentistry/healthcare-research) and Quality assessment scale

|  | source of information | Eligible for participants | Time for identifying patients | Consecutive  Subjects or not | Evaluate the subjective components of study | any assessments undertaken for quality assurance purpose | explain any patient exclusion from analysis | Assess or control confounding | How to handle the missing data | response rates and completeness of data collection | outcome of  follow-up |  |
| --- | --- | --- | --- | --- | --- | --- | --- | --- | --- | --- | --- | --- |
| AY et al. 2018 | 1 | 1 | 1 | 1 | 0 | 0 | 0 | 1 | 0 | 0 | 0 | 5 (moderate) |
| Kart et al. 2019 | 1 | 1 | 1 | 1 | 0 | 0 | 1 | 1 | 0 | 0 | 0 | 6  (moderate) |
| Benedetti et al. 2012 | 1 | 1 | 0 | 1 | 0 | 0 | 0 | 1 | 0 | 0 | 0 | 4 (moderate) |
| SELVI et al. 2012 | 1 | 1 | 0 | 1 | 0 | 0 | 0 | 0 | 0 | 0 | 0 | 3  (low) |
| Krah et al. 2012 | 1 | 1 | 0 | 1 | 0 | 0 | 0 | 1 | 0 | 1 | 0 | 5  (moderate) |
| Coban et al.2020 | 1 | 1 | 1 | 1 | 0 | 0 | 1 | 0 | 0 | 0 | 0 | 5 (moderate) |
| Carpenter et al.  2011 | 1 | 0 | 0 | 0 | 0 | 0 | 0 | 1 | 0 | 0 | 0 | 2  (low) |
| Semiz et al.  2014 | 1 | 1 | 1 | 1 | 0 | 0 | 0 | 1 | 0 | 1 | 1 | 7  (moderate) |

**3. 3. Summary of results of main meta-analyses and subgroup analyses**

Table 1. Summary of comparisons, sample sizes, effect sizes, P values and heterogeneity tests in different subgroup analyses.

Subgroup analysis of the meta-analysis of the association between CM and symptoms of OCD

| Subgroup | Studies  (n) | Sample Size  (n) | X^2^ | Heterogeneity  I^2^ | P | Effect Size | Summary Fisher's Z  95％CI | P |
| --- | --- | --- | --- | --- | --- | --- | --- | --- |
| Sample size |  |  |  |  |  |  |  |  |
| n≤150 | 4 | 783 | 9.14 | 67％ | 0.03 | 0.10 | -0.10-0.30 | 0.31 |
| n＞150 | 3 | 160 | 1.45 | 0％ | 0.48 | 0.09 | 0.01-0.17 | 0.03 |
| Measure |  |  |  |  |  |  |  |  |
| CTQ | 5 | 213 | 3.40 | 0％ | 0.49 | 0.07 | -0.00-0.14 | 0.05 |
| Non-CTQ | 2 | 730 | 3.12 | 68％ | 0.24 | 0.19 | -0.13-0.51 | 0.24 |

**4.Detailed results of subgroup analyses, sensitivity analyses (leave-one-out influence analyses), and publication bias:**

**4.1 subgroup analyses**

a. Subgroup by the assessment of childhood maltreatment:


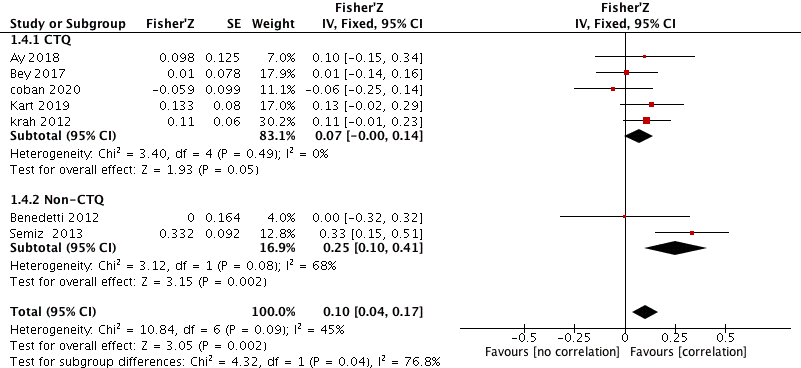


b. Subgroup by the sample size:


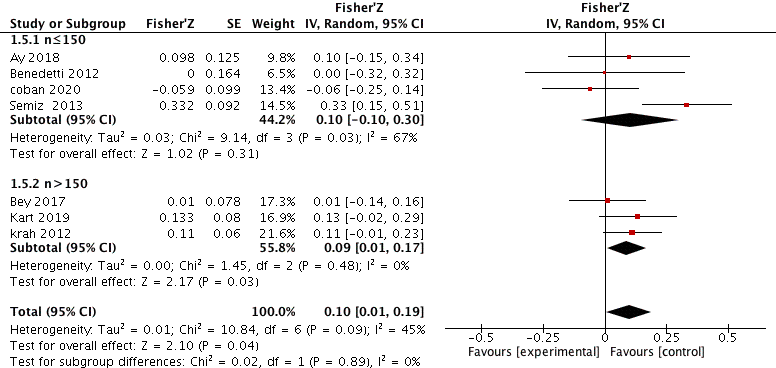


**4.2 Funnel plot**


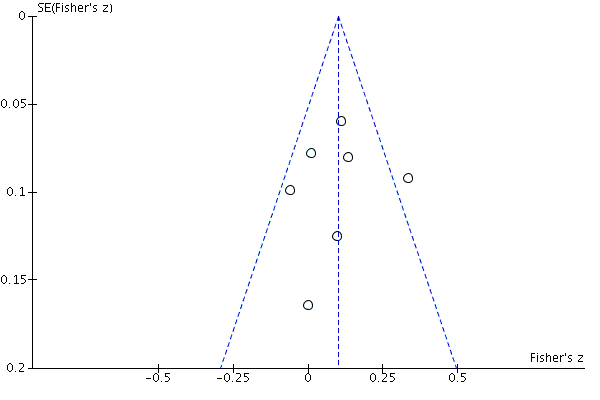


Fig 1. The Funnel plot of the meta-analysis of the association between CM and OCS severity


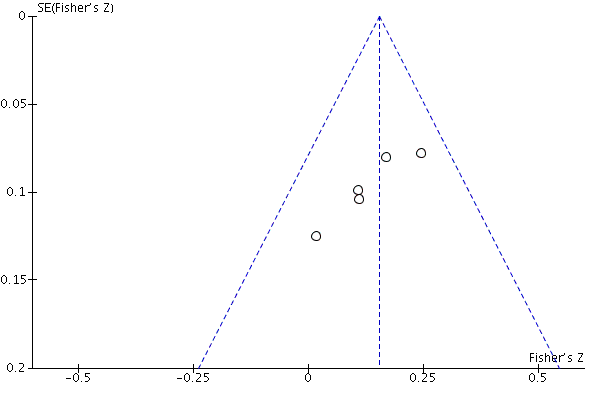


Fig 2. The Funnel plot of the meta-analysis of the association between CM and severity of depressive symptoms in OCD
